# Two-dimensional Brownian motion of active particle on superfluid helium surface

**DOI:** 10.1038/s41598-023-49672-3

**Published:** 2023-12-18

**Authors:** Roman E. Boltnev, Mikhail M. Vasiliev, Oleg F. Petrov

**Affiliations:** Joint Institute for High Temperatures of Russian Academy of Sciences, Izhorskaya Str. 13/2, Moscow, Russia 125412

**Keywords:** Colloids, Quantum fluids and solids

## Abstract

We report an experimental study of the 2D dynamics of active particles driven by quantum vortices on the free surface of superfluid helium at *T* = 1.45 К. The particle motion at short times (< 25 ms) relates to anomalous diffusion mode typical for active particles, while for longer times it corresponds to normal diffusion mode. The values of the rotational and translational kinetic energies of the particle allow to determine for the first time the intensity of the particle-vortex interaction and the dissipation rate of the vortex bundle energy. Strong bonding between a particle and a vortex is explained by coupling of normal and superfluid components.

## Introduction

Active particles are capable to acquire energy from their environment and use it for the self-propulsion. Collective motion of active particles is ubiquitous in nature at all scales. Collective phenomena with participation of such particles in dissipative non-equilibrium systems underlie self-organization processes and active matter^1^. Knowledge of the dynamics of active particle systems is of great importance in different fields of life and science. Collective motion of active particles may be described by a simple model in which stable directed motion of homogeneous active populations is caused by genuine physical interactions at the individual level^2^, while condensed matter physics concepts should be applied in study of evolution of biological systems^3^. Collective phenomena in live and artificial systems consisted of active Brownian particles may manifest as ordinary phase transitions^4,5^ or phase separation^6^. Artificial active materials are very promising for multiple engineering applications^7,8^.

One of the fundamental problems in modern physics is the role of quantum effects at the macroscopic scale (in the macroscopic world) and, in particular, in the evolution of macroscopic structures of matter^9^. Cryogenic colloid systems in the superfluid helium (He-II) may present a new class of active matter. Cooling liquid helium-4 down to the temperature *T*_λ_ = 2.176 K at the saturated vapor pressure brings it into a superfluid state related to the Bose–Einstein condensation of helium atoms occupying the lowest quantum state^10^. In this state liquid helium comprises a normal viscous fluid and an inviscid superfluid, which has no entropy. The phase transition is accompanied with self-organization of flows of the superfluid component^11^ carrying the angular momentum proportional to the temperature-dependent superfluid density *ρ*_*s*_^12^. Such flows of helium atoms bearing the same angular momentum, the quantum of circulation *κ* = *h/m* ≈ 1·10^−7^ m^2^/s, where *h* is Planck’s constant and *m* is mass of an atom ^4^He, form quantum vortices with an effective core radius, *a*_0_, equal to a few angstroms^13^. Quantum vortices, as topological defects of the order parameter (a macroscopic wave function) describing the system, are very stable and either end on interphase boundaries (linear vortices) or form closed curves (vortex rings) in the superfluid. Any “alien” particle (from the simplest ones, an electron and helium ions, to nanoclusters and microparticles) can be captured in quantum vortices due to Bernoulli pressure gradient in rotating superfluid^14^. Therefore, quantum vortices promote the aggregation of impurity particles into linear structures, along the vortex cores, in bulk^15^ and nanodroplets^16^ of superfluid helium-4. Motion of particles in superfluid helium can be governed by external magnetic or electric fields^17^, thermal counterflow^18,19^, interaction with quantum vortices^20,21^. A specific counterflow forms at the heated surface: the heat flux is carried away by the normal component, and, by conservation of mass, a superfluid flow develops in the opposite direction. At rather intense heat release on the surface the relative velocity between the superfluid and normal components may exceed the critical value. Then counterflow generates quantum vortices in superfluid helium bulk. Such a counterflow developed at the surface of a particle irradiated by intense laser light may result in formation of a turbulent vortex tangle around the particle^22,23^ and activation of a particle motion^24^. The driving role of quantum counterflow and turbulence in the active Brownian motion and evolution of structures consisting of superconducting grains levitating in He-II at temperatures below 2 K was revealed recently^21^. Very intense (increased by 6–7 orders of magnitude compared to the values from the Einstein formula) motion of grains was caused by quantum turbulence developed in counterflows around the grains heated with laser irradiation. The evolution of grain structures to a more ordered state with lower entropy, far from thermodynamic equilibrium, occurred due to the quantum mechanism of extremely high entropy loss in superfluid helium^21^.

Here we present the experimental study of active Brownian particle dynamics driven by quantum vortices on the free surface of superfluid helium at *T* = 1.45 К. The particle mean-square displacement and the energy dissipation in a vortex bundle were measured directly through the observation of translational and rotational motion of an active particle. The intense energy transfer from a vortex to the particle was explained by locking of the normal and superfluid components of He-II.

## Experiments

**Figure 1:**
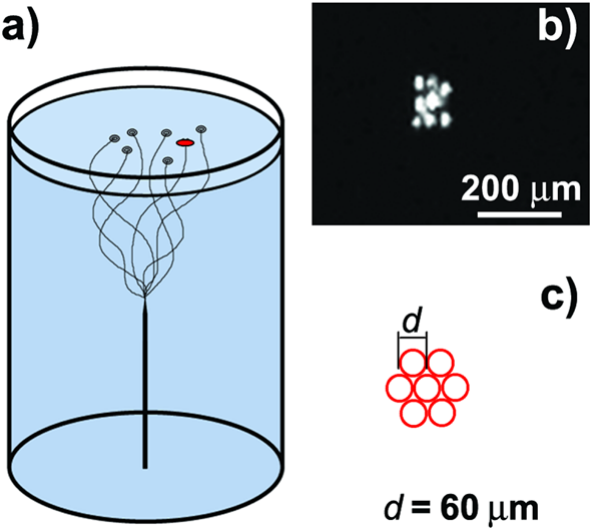
A particle on the free surface of He-II and its model: (**a**) Glass cell for observation of motion of the particle (shown as a red oval) driven by quantum vortices along the He-II surface; (**b**) Image of a particle in scattered light; (**c**) Simplified model of the particle used for estimations of its mass and inertial momentum: 6 glass microspheres surround the central one. The microspheres’ diameter is equal to 60 μm.

**Figure 2:**
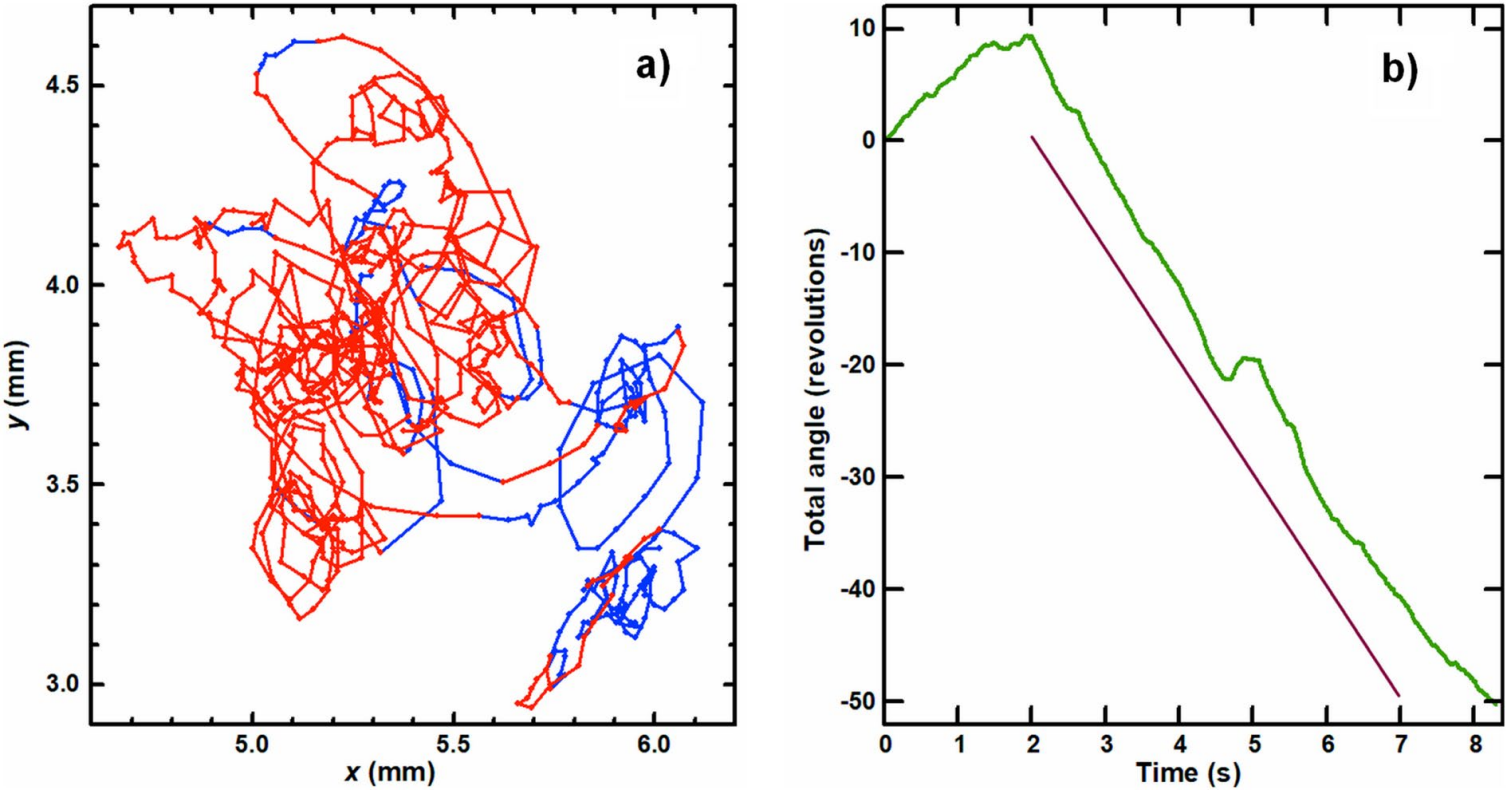
Particle motion: (**a**) Particle trajectory traced during 8.3 s at the rate100 fps (color points correspond to the particle positions, while red and blue colors reflect clockwise and counter-clockwise directions of particle rotation at these positions, respectively); (**b**) Time dependence of total rotation angle of the particle, the solid purple line corresponds to the angular velocity 10 rps.

A scheme of the experimental cell is shown in Fig. 1a. Some of residual quantum vortices^25^ pinned at a tungsten needle tip terminate at the free surface of He-II and form a vortex bundle in a cylindrical glass cell. To visualize the motion of the vortices we use a single particle with slightly positive buoyancy. More details on experimental setup and procedures can be found in “Methods”. The particle (cluster) was composed of 7–10 glass microspheres with the density a little lighter than 0.145 g/cm^3^, the liquid helium density at 1.45 K^26^. Such a particle localizes just below the He-II surface due to a balance of its positive buoyancy and surface tension of liquid helium^27,28^. Asymmetric shape of the particle along with its large size ≤ 0.2 mm, Fig. 1b, allowed us to observe its rotational motion along with the translational one. The upper end of a vortex traps the particle (Fig. 1) and they move together along the He-II surface. The particle trajectory along the He-II surface recorded at the rate 100 frames per second (fps) is shown in Fig. 2. The mean velocity of the particle is found unexpectedly high, ≈ 6 mm/s, or about 4 orders of magnitude higher than the velocity value estimated for thermal fluctuations of liquid helium at 1.45 K. This fact is explained by energy transfer from moving quantum vortices to the particle.

## Results and discussion

The trajectory fragments corresponding to the clockwise/counterclockwise rotation of the particle are marked with red/blue color, respectively, in Fig. 2a. We relate the direction changes of the particle rotation to its jumps from one vortex to another (with opposite charges).

**Figure 3:**
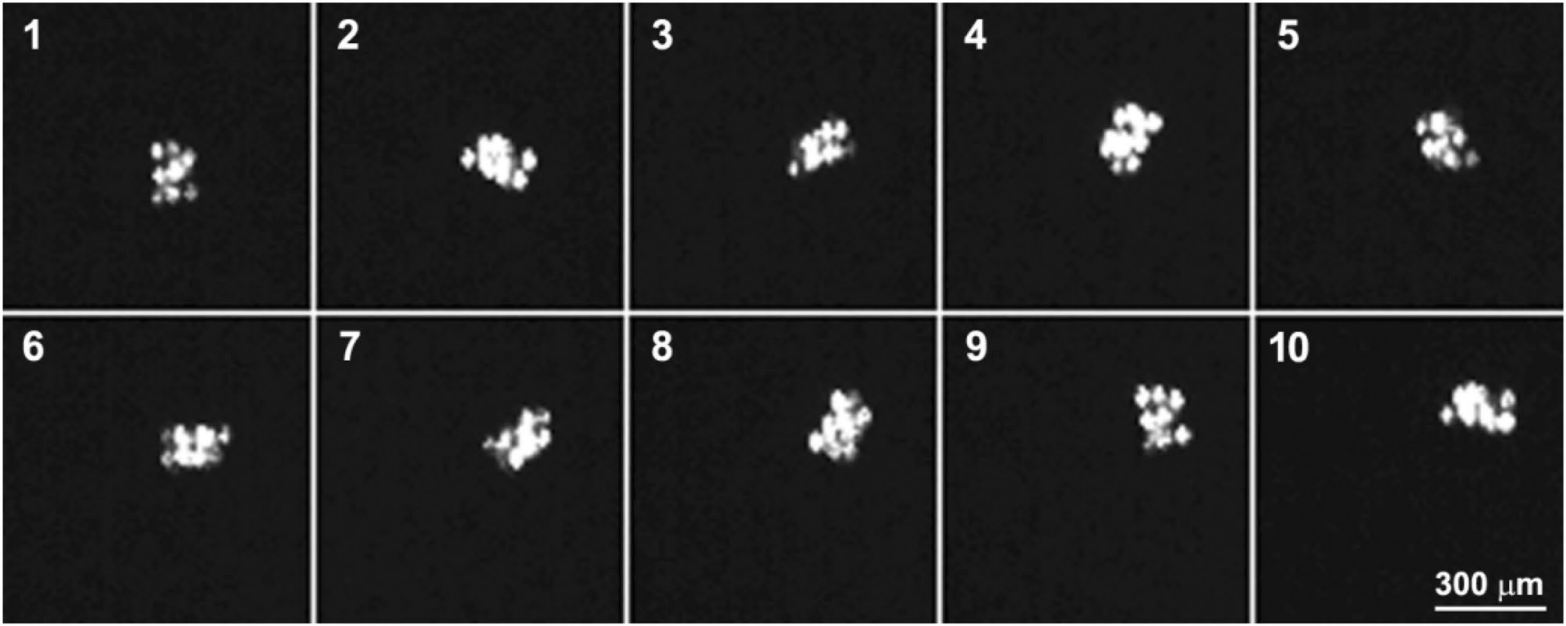
Motion of the spinning particle along the surface of He-II recorded at the rate 2000 fps. A time interval between the frames shown is 10 ms.

From overlapping the trajectories of vortices with opposite charges we can conclude that there are no static positions of the vortices pinned at the He-II surface. Video of a spinning particle recorded at the rate 100 fps can be found in Supplementary Materials. One can see the motion of the particle is a combination of translational and rotational motions. The switching of the rotation direction usually occurs within 0.05–0.2 s, while the residence time of the particle at a vortex and the time of transit between vortices, are equal to ≈ 1 s and ≤ 0.1 s, respectively. Rather frequent and fast transitions of the particle between vortices point to short intervortex distances ~ 0.1 mm. The angular velocity of the particle is not a constant value (Fig. 2b): but its mean value is close to ≈ 10 revolutions per second (rps), while a maximal value reaches 20 rps. Some fragments of the particle trajectory seem to be straight and the particle shape is smeared due to low rate of the video. A real trajectory of the particle was recorded at the higher rate 2000 fps. It was found that the translational velocity of the particle can reach 20 mm/s while its mean value was equal to ≈ 6.9 mm/s. The particle rotation is shown in Fig. 3. The frames shown are separated by a time interval 10 ms. The particle consists of 9 glass microspheres bound together with van der Waals interaction^29,30^. Its image shape changes during rotation because of laser illumination from one side and is reproducible approximately every 80 ms.

**Figure 4:**
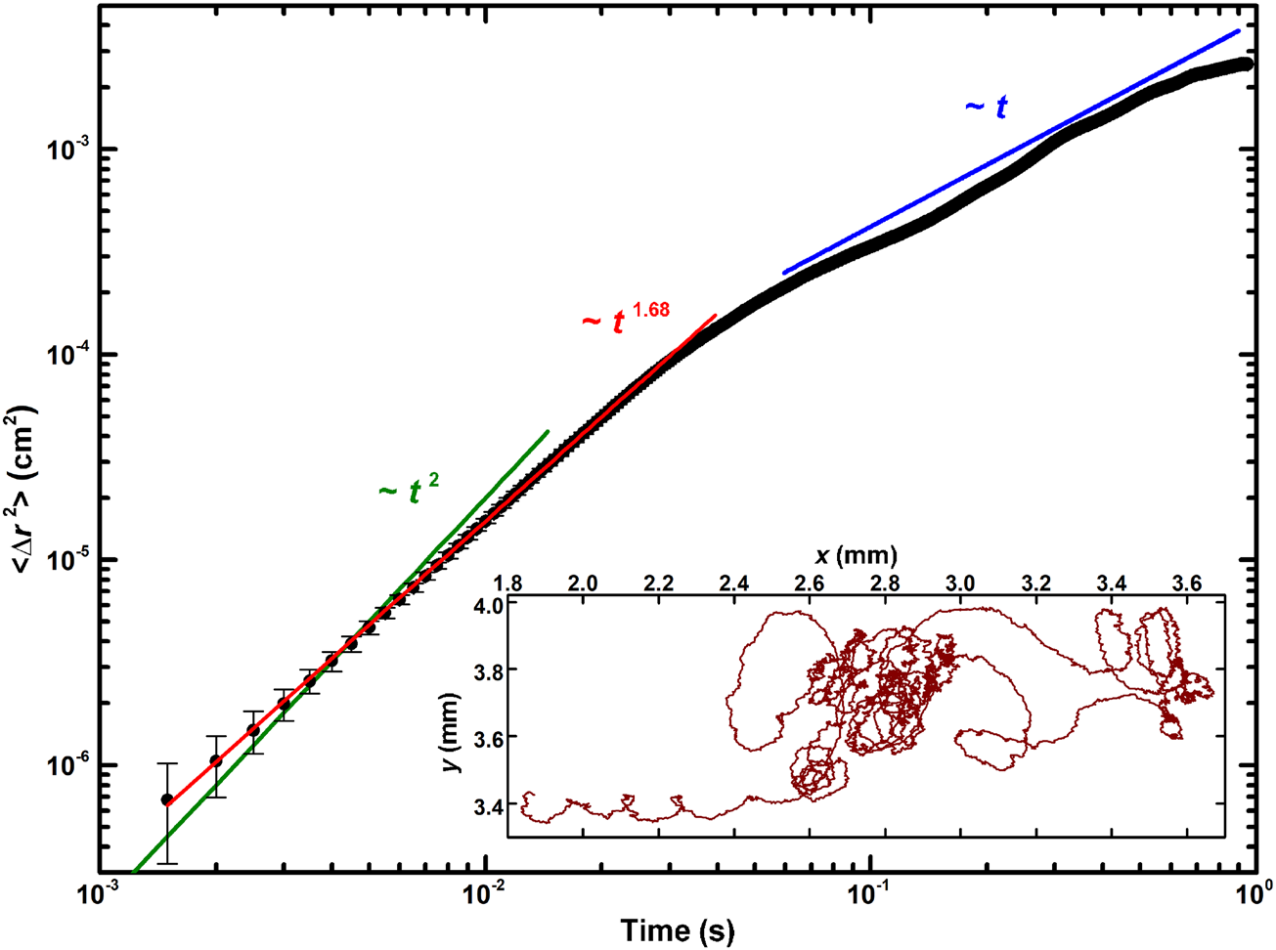
Temporal dependence of the mean-squared displacement for the active particle (double-logarithmic plot). The blue and green lines indicate the slopes, *α*, equal to 1 and 2, respectively. The red line corresponds to the fit of the particle motion at short times (< 25 ms). The inset shows the particle trajectory recorded at the rate 2000 fps. Error bars indicate the spatial resolution of the detection system.

The particle motion was analyzed by calculation of the time dependence of its mean-square displacement < Δ*r*^2^(*t*) > and the Peclet number, which reflects the relative importance of directed motion versus diffusion for an active Brownian particle^1^. The time dependence of the mean-square displacement for the particle as well as its trajectory captured at the rate 2000 fps are shown in Fig. 4 and the inset, respectively. The figure shows also blue and green lines corresponding to different modes of Brownian motion < Δ*r*^2^(*t*) >  ~ *t*^*α*^: *α* = 1 for normal diffusion, and *α* = 2 for ballistic mode. Intermediate values of *α* are related to anomalous diffusion mode. The experimental curve is very well fitted with *α* = 1.68 at short times, *t* < 25 ms (red line in Fig. 4). Thus, the particle motion at short times relates to anomalous diffusion mode (or superdiffusion) typical for active particles, while for longer times it corresponds to normal diffusion mode.

To obtain the Peclet number we use the equation from^31^

$$Pe\sim v \cdot d/D$$

where *v* and *d* are the velocity and diameter of the particle, ≈ 10 mm/s and ~ 0.1 mm, respectively, *D* is its diffusion coefficient. The obtained value, *Pe* ≈ 12, does reflect domination of directed motion of the particle versus its diffusion (for diffusion *Pe* ≤ 1).

The effective diffusion coefficient, *D*, for the diffusive mode was found equal to 8.3·10^−4^ cm^2^/s or ≈ 0.83*κ*, in good agreement with the experimental value 0.3*κ* measured for solid deuterium microparticles trapped by quantum vortex^32^ and the theoretical estimations 0.53κ^33^ made for diffusion of quantum vortices in bulk He-II at *T* = 0 K. It is interesting to compare the experimental value of the diffusion coefficient, 8.3·10^−4^ cm^2^/s, with the theoretical one, ≈ 1·10^−10^ cm^2^/s, obtained for a Brownian particle following to the Einstein formula^34^

$$D_{T} = k_{B} \cdot T/(3\uppi \cdot \eta \cdot d)$$

where *k*_B_ is the Boltzmann constant and *η* is the viscosity of liquid helium, *η* ≈ 1.4·10^−6^ Pa·s at *T* = 1.45 K^26^. Exclusively low viscosity of He-II breaks the ultimate demands on sub-ångström spatial and sub-microsecond temporal resolutions for observation ballistic mode of Brownian motion in classical liquids, for example, in water^35^. The momentum relaxation time, *τ*_*p*_, for a single glass microsphere is equal to ≈ 23 ms. Nevertheless, the particle motion at short time scales (Fig. 4), is not ballistic with *α* = 1.68, but this value is in good agreement with the *α* values within a range 1.6–1.8 experimentally observed for motion of hydrogen^36^ and deuterium^32^ particles driven by quantum vortices generated in thermal counterflow at temperatures 1.7–2 K. Recently a numerical study of quantum vortex diffusion in turbulence induced by a random tangle of vortices demonstrated that anomalous diffusion takes a place at short times and transits to normal one at longer times ~ 0.1 s^37^.

Let us evaluate the energy and the total angular momentum^13^ of a quantum vortex pinned to the particle, suggesting the vortex radius value, *R*_0_ ≈ 0.2 mm, two times larger than the particle diameter:

$$E_{v} = H_{0} \cdot \rho_{s} \cdot \kappa^{2} /(4\uppi) \cdot \ln (R_{0} /a_{0} ) \approx 6.1 \cdot 10^{ - 14} \;{\text{J}}$$

$$L_{v} = H_{0} \cdot \rho_{s} \cdot \kappa \cdot R_{0}^{2} /2 \approx 2.6 \cdot 10^{ - 15} \;{\text{kg}}\;{\text{m}}^{2} /{\text{s}}$$

where *H*_0_ ≈ 40 mm is the vortex length, *a*_0_ = 0.1 nm—the vortex core radius, and *ρ*_*s*_ ≈ 132 kg/m^3^—the superfluid density of He-II at 1.45 K^26^. The vortex radius value was estimated from the experimental fact that the particle rotation was interrupted only for very short periods ≈ 0.1 s. Rotation of the particle is possible only if it is captured by one quantum vortex. Pinning to the particle of a bundle of two and more vortices will result in stronger binding and the cessation of the particle rotation^38^. Therefore, the vortex radius has to be larger than the particle radius (≈ 0.1 mm), but less than 0.1·7/2 ≈ 0.35 mm, where 0.1 s is the transit time of the particle between neighbor vortices and 7 mm/s is the mean particle velocity. Hence, we can evaluate the intervortex distance equal to ≈ 0.4 mm and the vortex radius as 0.2 mm, keeping in mind that the transit time does also include switching of the particle rotation direction.

Now we estimate the energetic parameters of the particle interaction with quantum vortices. The kinetic energy of translational motion of the particle, corresponding to its average velocity 7 mm/s, is of the order of 10^−15^ J. To determine the angular momentum of the particle, *L*_*p*_, we calculate its moment of inertia, *I*_*p*_ ≈ 4.2·10^−19^ kg·m^2^, following a simplified model shown in Fig. 1c. Keeping in mind the mean value of the particle angular velocity 10 rps, we find *ω* = 20π s^−1^ and *L*_*p*_ ≈ 2.6·10^−17^ kg·m^2^/s or 4·10^16^ h. Therefore, approximately 10^17^ helium atoms have to cease their motion in a quantum vortex to switch the direction of the particle rotation. The rotating particle has the average kinetic energy of *K*_*p*_ ~ 10^−15^ J. Then the rate of the energy transfer from a quantum vortex to the particle should be of the order of 10^−14^ W, because the switching of a rotation direction takes the time ≈ 0.1 s. It is a rather strong binding which can be explained exclusively by coupling of normal and superfluid components observed at Reynolds numbers > 10^39^. The normal and superfluid components move independently only at very low velocities. At higher velocities the normal component velocity field is disturbed by friction on cores of quantum vortices and both fluids begin to move together at Reynolds numbers ~ 10. For the particle radius, *R*_p_, 0.1 mm and *ω* = 20π s^−1^ we get the Reynolds number value ≈ 66 following to the formula

$${\rm Re} = \omega \cdot R_{\rm p}^{2} /\upnu$$

where is the kinematic viscosity of He-II equal to 9.5·10^−9^ m^2^/s at 1.45 K^26^. Possibly, the same effect was responsible for the rotation of elongated dust particles moving along quantum vortices in bulk He-II^40^.

In summary, we observed two-dimensional Brownian motion of active particles induced by quantum vortices in superfluid helium. The particle motion corresponded to anomalous diffusion mode typical for active particles at short times (< 25 ms) and to normal diffusion mode for longer times. The rotational energy and angular momentum of the particle captured by a quantum vortex were determined as ~ 10^−15^ J and ≈ 2.6·10^−17^ kg·m^2^/s, correspondingly, in good accordance with the energy and total angular momentum of the quantum vortex were estimated as ≈ 6.1·10^−14^ J and ≈ 2.6·10^−15^ kg·m^2^/s, respectively. It was found that interaction of a particle with quantum vortices reveals a very efficient energy transfer from a vortex tangle to a particle due to locking of the normal and superfluid components of superfluid helium.

## Methods

**Figure 5:**
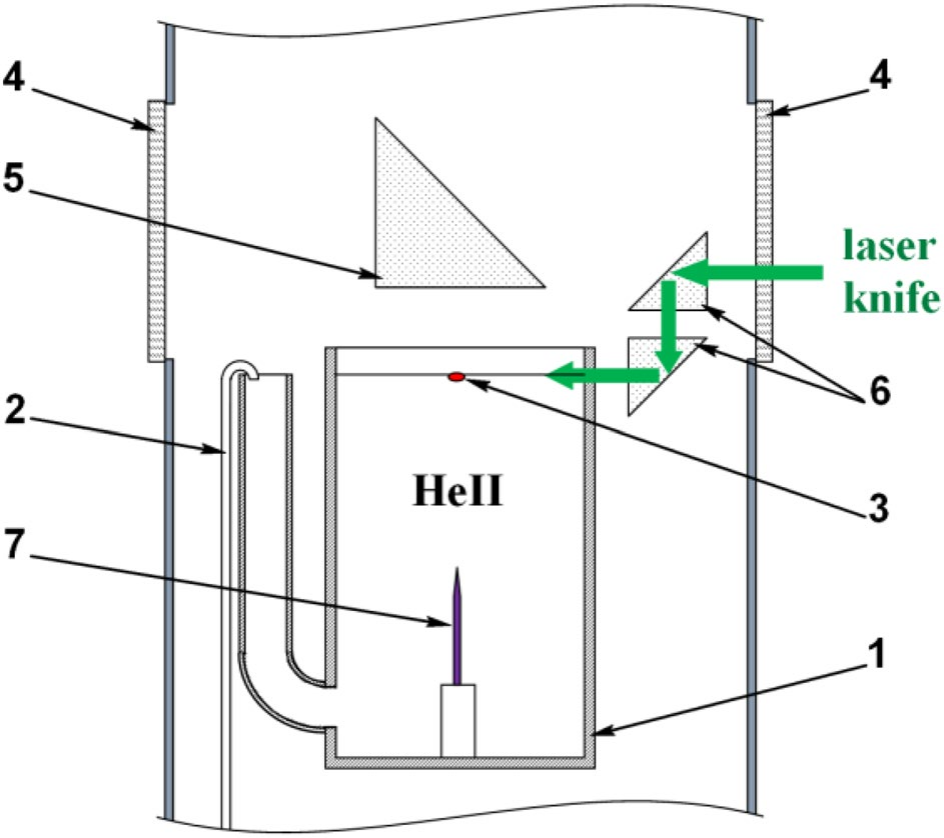
Scheme of a cryogenic part of the experimental setup: 1—glass cell with the lateral tube; 2—capillary of thermomechanical pump; 3—a particle at the free surface of He-II in the cell; 4—optical windows of the cryostat; 5—a prism for particle observation; 6—a prism periscope; 7—tungsten needle.

The experiments were carried out in an optical cryostat Janis SVT-200 equipped with windows on four sides. The temperature of superfluid helium inside the cryostat was measured using a calibrated thermometer and kept equal to 1.45 K by the vapor pressure regulation. A scheme of a cryogenic part of the experimental setup is shown in Fig. 5. A cylindrical glass cell with the inner diameter 20 mm, the cell height 65 mm (position 1 in Fig. 5) was filled with superfluid helium by a thermomechanical pump (pos. 2 in Fig. 5) through a lateral glass tube. The level of He-II in the cell (3 mm below the cell edge) was determined by the lateral tube height and was kept constant due to continuous work of the pump located in the He-II bath. A tungsten needle was fixed at the center of the cell bottom. Its tip was about 36 mm below the He-II surface. Some of residual vortices formed upon filling the cell with He-II remain pinned at the needle tip and terminate at the free surface of He-II (Fig. 1). Such geometry allows to obtain a tangle of vortices which can trap a particle floating at the surface. Motion of the particle was recorded through cryostats windows and a quartz prism (positions 4 and 5 in Fig. 5, correspondingly) by a high-speed digital camera IDT MotionPro Y3 at the rates from 100 to 2000 fps. The duration of continuous video recording of particles at the rate of 2000 fps was limited by 5 s due to the memory buffer volume of a high-speed video camera (10,000 frames 512 × 512 pixels). We have analyzed 5 particle trajectories recorded during 5 s to determine the slope value *α* = 1.68 ± 5 (s. d.) of the mean-squared displacement at times shorter than 25 ms. A lens Sigma 105 mm f/2.8 EX DG Macro provided the spatial resolution about of 12 μm (85 pixels/mm). A continuous-wave laser knife (thickness, 250–300 μm and width, 12 mm) was guided along with the He-II surface by a prism periscope (pos. 6 in Fig. 5). Keeping in mind the reflections from glass surfaces, the laser (power up to 100 mW at 532 nm) the maximal power density was ≈ 1 W/cm^2^.

**Figure 6:**
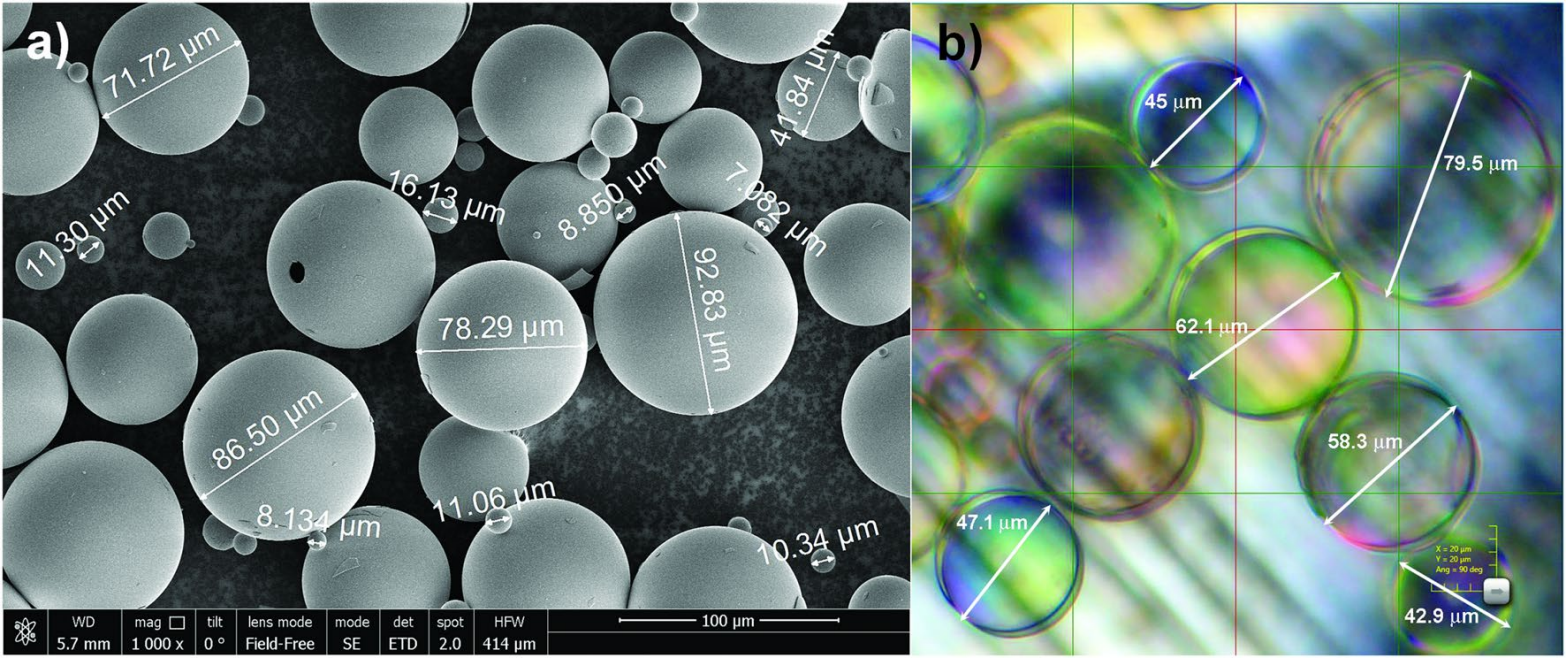
Micrographs of glass microspheres M3 A16/500: (**a**) scanning electron microscope FEI Nova NanoSEM 650, gain ×1000; (**b**) optical microscope DSX-1000, gain ×1200.

Polydisperse hollow microspheres with methacrylato chromic chloride surface treatment (Glass Bubbles Floated A16/500 from 3M) with sizes ranging from 8 to 120 μm and densities ranging from 0.14 to 0.18 g/cm^3^ were used in the experiments (Fig. 6). The mean diameter and the wall thickness of the microspheres were about of 60 μm and 1 μm, respectively. The microspheres were completely transparent for visible light (Fig. 6b).

Some hundred of microspheres were loaded onto the cell bottom before experiments. Some of microspheres have the density less than 0.145 g/cm^3^, the density of He-II at 1.45 K. Thus, if the average density of a particle consisting of 7–10 glass microspheres, bound together due to van der Waals interaction, was less than 0.145 g/cm^3^ it could come up to the surface after light knocking the cryostat. The inertia moment of particle, *I*_*tr*_, was estimated accordingly to Huygens–Steiner theorem for its simplified flat model, shown in Fig. 1c. All seven microspheres with the same diameter of *d* = 60 μm lie in the same plane. The distances between the centers of the central microsphere and the outer ones are equal to 60 μm. The inertia moment about the axis the axis perpendicular to the particle plane.

Video records were reviewed and preliminary handled with software Motion Studio. The particle motion was processed and analyzed with the specially developed software “Plasma”.

It should be noted that the measurements were carried out in the stationary conditions when the dissipation rate from a vortex tangle was approximately equal to the energy-injection rate: the vortex motion induced by its interaction with others vortices as well as with excitations of liquid helium (mechanical vibrations, heat releases and the like) remained constant during the experiment.

### Supplementary Information

Supplementary Video 1.

Supplementary Information 1.

## Data Availability

The datasets analyzed during the current study are available from the corresponding author on reasonable request.

## References

[1] Bechinger C (2016). Active particles in complex and crowded environments. Rev. Mod. Phys..

[2] Bricard A, Caussin J-B, Desreumaux N, Dauchot O, Bartolo D (2013). Emergence of macroscopic directed motion in populations of motile colloids. Nature.

[3] Goldenfeld N, Woese C (2011). Life is physics: Evolution as a collective phenomenon far from equilibrium. Annu. Rev. Condens. Matter Phys..

[4] Petrov OF (2015). Solid–hexatic–liquid transition in a two-dimensional system of charged dust particles. EPL.

[5] Vasilieva EV, Petrov OF, Vasiliev MM (2022). Laser-induced melting of two-dimensional dusty plasma system in RF discharge. Sci. Rep..

[6] Cates ME, Tailleur J (2015). Motility-induced phase separation. Annu. Rev. Condens. Matter Phys..

[7] Paxton WF (2004). Catalytic nanomotors: Autonomous movement of striped nanorods. J. Am. Chem. Soc..

[8] Bayındır L (2016). A review of swarm robotics tasks. Neurocomputing.

[9] Fröwis F, Sekatski P, Dür W, Gisin N, Sangouard N (2018). Macroscopic quantum states: Measures, fragility, and implementations. Rev. Mod. Phys..

[10] London F (1938). The λ-phenomenon of liquid helium and the Bose–Einstein degeneracy. Nature.

[11] Zurek WH (1985). Cosmological experiments in superfluid helium?. Nature.

[12] Reppy JD, Depatie D (1964). Persistent currents in superfluid helium. Phys. Rev. Lett..

[13] Feynman RP, Gorter CJ (1955). Application of quantum mechanics to liquid helium. Progress in Low Temperature Physics.

[14] Hills RN, Roberts PH (1978). Healing and relaxation in flows of helium II. III. Pure superflow. J. Phys. C.

[15] Gordon EB, Nishida R, Nomura R, Okuda Y (2007). Filament formation by impurities embedding into superfluid helium. JETP Lett..

[16] Ulmer A (2023). Generation of large vortex-free superfluid helium nanodroplets. Phys. Rev. Lett..

[17] Golov AI, Walmsley PM, Tompsett PA (2010). Charged tangles of quantized vortices in superfluid ^4^He. J. Low Temp. Phys..

[18] Murakami M, Ichikawa N (1989). Flow visualization study of thermal counterflow jet in He II. Cryogenics.

[19] Kolmakov GV, Aranson IS (2021). Superfluid swimmers. Phys. Rev. Res..

[20] Paoletti MS, Fiorito RB, Sreenivasan KR, Lathrop DP (2008). Visualization of superfluid helium flow. J. Phys. Soc. Jpn..

[21] Petrov OF, Boltnev RE, Vasiliev MM (2022). Experimental evolution of active Brownian grains driven by quantum effects in superfluid helium. Sci. Rep..

[22] Inui S, Tsubota M (2020). Spherically symmetric formation of localized vortex tangle around a heat source in superfluid ^4^He. Phys. Rev. B.

[23] Moroshkin P, Leiderer P, Kono K, Inui S, Tsubota M (2019). Dynamics of the vortex-particle complexes bound to the free surface of superfluid helium. Phys. Rev. Lett..

[24] Inui S, Tsubota M, Moroshkin P, Leiderer P, Kono K (2019). Dynamics of fine particles due to quantized vortices on the surface of superfluid ^4^He. J. Low Temp. Phys..

[25] Awschalom DD, Schwarz KW (1983). Observation of a remanent vortex-line density in superfluid helium. Phys. Rev. Lett..

[26] Donnelly RJ, Barenghi CF (1998). The observed properties of liquid helium at the saturated vapor pressure. J. Phys. Chem. Ref. Data.

[27] Levchenko AA, Lebedeva EV, Mezhov-Deglin LP, Pelmenev AA (2019). Self-organization of neutral particles on the surface of superfluid He II. Low Temp. Phys..

[28] Dyugaev AM, Lebedeva EV (2017). Surface microparticles in liquid helium. Quantum Archimedes’ principle. JETP Lett..

[29] Bewley GP, Sreenivasan KR, Lathrop DP (2008). Exp. Fluids.

[30] Boltnev RE, Bykhalo IB, Krushinskaya IN (2022). Impurity systems in condensed helium-4. J. Low Temp. Phys..

[31] Dombrowski C, Cisneros L, Chatkaew S, Goldstein RE, Kessler JO (2004). Self-concentration and large-scale coherence in bacterial dynamics. Phys. Rev. Lett..

[32] Tang Y, Bao S, Guo W (2021). Superdiffusion of quantized vortices uncovering scaling laws in quantum turbulence. Proc. Natl Acad. Sci. USA.

[33] Rickinson E, Parker NG, Baggaley AW, Barenghi CF (2019). Inviscid diffusion of vorticity in low-temperature superfluid helium. Phys. Rev. B.

[34] Einstein A (1905). Uber die von der molecularkinetischen theorie der warme geforderte bewegung von in ruhenden flussigkeiten suspendierten teilchen. Ann. Phys..

[35] Kheifets S, Simha A, Melin K, Li T, Raizen MG (2014). Observation of Brownian motion in liquids at short times: Instantaneous velocity and memory loss. Science.

[36] Chen L, Maruyama T, Tsuji Y (2022). Statistical properties of Lagrangian trajectories of small particles in superfluid ^4^He. J. Low Temp. Phys..

[37] Yui S, Tang Y, Guo W, Kobayashi H, Tsubota M (2022). Universal anomalous diffusion of quantized vortices in ultraquantum turbulence. Phys. Rev. Lett..

[38] Giuriato U, Krstulovic G, Nazarenko S (2020). How do trapped particles interact with and sample superfluid vortex excitations?. Phys. Rev. Res..

[39] Donnelly RJ (1993). Quantized vortices and turbulence in helium II. Annu. Rev. Fluid Mech..

[40] Jin D, Maris HJ (2011). A study of the motion of particles in superfluid helium-4 and interactions with vortices. J. Low Temp. Phys..

