# Supplementary material for: Two-dimensional Brownian motion of active particle on superfluid helium surface
Source: Sci Rep. 2023 Dec 18;13:22538. doi: 10.1038/s41598-023-49672-3 (PMC10728076; doi:10.1038/s41598-023-49672-3)
Supplement: Supplementary file 2 — Supplementary Information 1. [file 41598_2023_49672_MOESM2_ESM.docx]

**Supplementary Information**

File S1movie.avi contains a video of a spinning particle recorded at the rate 100 fps.
